# Disruption of NBS1/MRN Complex Formation by E4orf3 Supports NF-κB That Licenses E1B55K-Deleted Adenovirus-Infected Cells to Accumulate DNA>4n

**DOI:** 10.1128/spectrum.01881-21

**Published:** 2022-01-12

**Authors:** Nujud Almuzaini, Madison Moore, Marjorie Robert-Guroff, Michael A. Thomas

**Affiliations:** a Department of Biology, College of Arts and Sciences, Howard University, Washington, D.C., USA; b Section on Immune Biology of Retroviral Infection, Vaccine Branch, National Cancer Institute, National Institutes of Health, Bethesda, Maryland, USA; Barnard College, Columbia University

**Keywords:** ATM, DNA damage, DNA>4n, E1B55K-deleted Ad, E4orf3, MRN, NBS1, NF-kB, adenovirus

## Abstract

Cells increase their DNA content greater than the G2/M (DNA > 4n) phases along the path to cancer. The signals that support this increase in DNA content remain poorly understood. Cells infected with adenovirus (Ad) similarly develop DNA > 4n and share a need to bypass the DNA damage response (DDR) signals that trigger cell cycle arrest, and/or cell death. Ads with deletion in early region 1B55K (Δ*E1B* Ad) are oncolytic agents that are currently being explored for use in vaccine delivery. Interestingly, they promote higher levels of DNA > 4n than Ads that contain E1B55K. Existing in these and almost all Ads that are being explored for clinical use, is early region 4 (E4). The Ad E4 open reading frame 3 (E4orf3) is a viral oncogene that interferes with the ability of cells to respond to DNA damage by disrupting MRN complex formation. Our study reveals that E4orf3 is required for the enhanced fraction of Δ*E1B* Ad-infected cells with DNA > 4n. For that reason, we explored signaling events mediated by E4orf3. We found that in Δ*E1B* Ad-infected cells, E4orf3, as reported by others, isolates NBS1 in nuclear dots and tracks. This allows for elevated levels of phosphorylated ATM that is linked to transcriptionally active NF-κB. Pharmacological inhibition of NF-κB reduced the fraction of Δ*E1B* Ad-infected cells with DNA > 4n while pharmacological inhibition of ATM reduced the levels of nuclear NF-κB and the fraction of Δ*E1B* Ad-infected cells with DNA > 4n and increased the fraction of dead or dying cells with fragmented DNA. This ability of E4orf3 to disrupt MRN complex formation that allows cells to bypass the cell cycle, evade death, and accumulate DNA > 4n, may be linked to its oncogenic potential.

**IMPORTANCE** Genome instability, a hallmark of cancer, exists as part of a cycle that leads to DNA damage and DNA > 4n that further enhances genome instability. Ad E4orf3 is a viral oncogene. Here, we describe E4orf3 mediated signaling events that support DNA > 4n in Δ*E1B* Ad-infected cells. These signaling events may be linked to the oncogenic potential of E4orf3 and may provide a basis for how some cells survive with DNA > 4n.

## INTRODUCTION

Besides the occurrence in early development (1) and in some specialized cells (2), cells increase their DNA content beyond the G2/M cell cycle phases on the path to cancer (3, 4). This increase in DNA content allows the cells to build tolerance for DNA damage and promotes genome instability (3, 5). One of the factors that guards access to cellular DNA content greater than the G2/M phases (DNA > 4n) is the MRE11-RAD50-NBS1 (MRN) complex. In response to double-strand breaks (DSB) the MRN complex guides the Serine/Threonine protein kinase ataxia-telangiectasia mutated (ATM) and ATM-and-Rad3-related (ATR) protein kinase (6–8) through a cascade of events that is collectively termed the DNA damage response (DDR) (9). It is the job of the DDR machinery to identify the damage, arrest the cell cycle, repair the damage (10) and when all else fails, initiate cell death (11).

Adenovirus (Ad) early region 1A (E1A) induces oncogenic transformation through interaction with p300/CBP, TRRAP/p400 multiprotein complex, and the retinoblastoma (pRb) family of proteins (12–15). These interactions are among the many that instruct DNA > 4n in E1A-expressing cells (16). Besides DNA > 4n, E1A-containing Ad-infected cells also display other signs that precede genome instability, including double- and single-strand DNA breaks (17–19). Some or all of these activities stimulate DDR proteins and/or DDR activities that limit Ad progeny production (20). Because of this, Ad encodes 3 gene-products that act to nullify the effects of the induced DDR: early region 1B55K (E1B55K), early region 4 open reading frame 6 (E4orf6), and early region 4 open reading frame 3 (E4orf3) that separately have been shown to help E1A to more efficiently transform cells (21–23). Ad E1B55K forms a complex with E4orf6 and together with other cellular factors induces the degradation of numerous DDR proteins, including MRE11 (24). Independent of E1B55K and E4orf6, Ad E4orf3 inactivates the MRN complex by relocalizing and/or isolating MRE11 and NBS1 into nuclear dots and tracks (25–27). Thus, Ad disrupts MRN complex formation by disparate means. Because in some cell systems, disruption of MRN complex formation leads to DNA > 4n (4, 28) it has remained possible that in Ad-infected cells E4 products contribute to DNA > 4n. The Ad E4 contains E4orf1, E4orf2, E4orf3, E4orf4, E4orf6, and E4orf6/7. To test the idea that in Ad-infected cells E4 products contribute to DNA > 4n, we first created wild type Ad with deletions in E4orf1, E4orf1-2, E4orf1-3 and E4orf1-4 (29). All these Ad with functional E1B55K and E4orf6 promoted similar levels of cells with DNA > 4n independent of the other E4 genes (29). This we surmise may have been because the activities undertaken by the E1B55K/E4orf6 complex with regard to DNA > 4n supersede those carried out by the remaining E4 products. Indeed, E1B55K-deleted (Δ*E1B*) Ads promote DNA > 4n in a larger fraction of cells than E1B55K-containing Ads (30).

In this study, we show that E4orf3 is required for the enhanced levels of Δ*E1B* Ad-infected cells with DNA > 4n. We explored signaling events mediated by E4orf3 and now report that in Δ*E1B* Ad-infected cells, disruption of MRN complex formation associates with transcriptionally active NF-κB that is linked to phosphorylated ATM. In contrast, in *E4orf3*-deleted Δ*E1B* Ad infections, the fraction of cells with DNA > 4n, levels of nuclear NF-κB, and levels of phosphorylated ATM were significantly reduced. Pharmacological inhibition of NF-κB reduced the fraction of Δ*E1B* Ad-infected cells with DNA > 4n while pharmacological inhibition of ATM reduced the levels of nuclear NF-κB and the fraction of Δ*E1B* Ad-infected cells with DNA > 4n but increased the fraction of cells with fragmented DNA.

## RESULTS

### Ad infection leads to an accumulation of cells with DNA > 4n.

**FIG 1:**
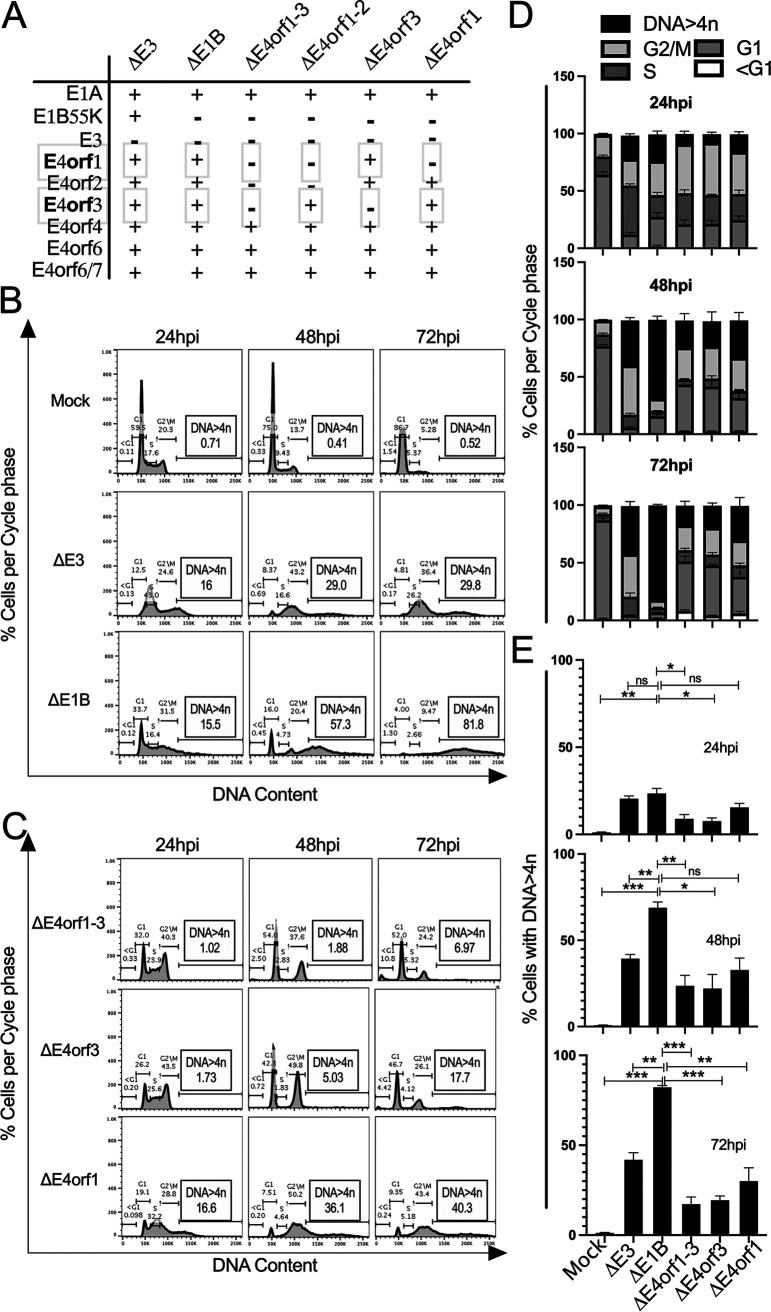
Ad E4 products support DNA > 4n in infected cells. (A) The Ad used in this study are listed on the top horizontal axis and the genes of interest on the vertical axes. Their status is indicated by + or − (B) A549 cells were mock infected, or infected with *ΔE3* or *ΔE1B* Ad; (C) *ΔE4orf1-3*, *ΔE4orf3* and *ΔE4orf1* Ad. In B and C, the cells were fixed at 24, 48, and 72 hpi, and exposed to PI/RNase staining solution. The DNA cell cycle profiles were interrogated by flow cytometry and analyzed with FlowJo (Becton, Dickinson). Analysis of the level of cells per cycle phases (D) and DNA > 4n (E) over time. The graphs illustrate mean ±SEM values of *n* = 6 independent experiments. *P values* were obtained using two-way ANOVA where *, *P* < 0.05; **, *P* < 0.01; ***, *P* < 0.001.

In Fig. 1A we provide a list of the viruses used within this study. We (29) and others (16, 30) have reported that post Ad infection cells accumulate DNA > 4n. Here, A549 cells were either mock-infected, or infected with a phenotypic wild type Ad (Δ*E3 Ad*), or a *E1B55K*-deleted Ad (31) (Δ*E1B* Ad), for 24, 48, and 72 h (Fig. 1A and B). The cells were interrogated by flow cytometry after being stained in an RNase solution containing propidium iodide (PI) as described in the Methods section. As expected, mock-infected cells exhibited extremely low levels of DNA > 4n (Fig. 1B, row 1). In contrast, 24 h postinfection (hpi), 16% of the Δ*E3* Ad-infected cells contained DNA > 4n. This percentage increased over time (from 16% to 29% by 72 hpi) (Fig. 1B, row 2). In comparison, by 72 hpi 81.8% of the cells that were infected by the Δ*E1B* Ad exhibited various levels of DNA > 4n (Fig. 1B, row 3). These results recapitulate the finding of a previous report (30) and suggest that Ad E1B55K (most likely in complex with E4orf6) acts to limit DNA > 4n in Ad-infected cells. This supports our notion that the activities undertaken by the E1B55K/E4orf6 complex with regard to DNA > 4n supersede those carried out by the E4 products and explain why we did not detect changes in DNA > 4n in E4orf1- to E4orf4-deleted wild type Ad (29).

### E4orf1 and E4orf3 support DNA > 4n in ΔE1B Ad infected cells.

To determine whether Ad E4 products impact levels of DNA > 4n observed in infections with Δ*E1B* Ad, cells were infected with Δ*E1B* Ads that contained various E4 gene deletions as indicated in Fig. 1C. The DNA cell cycle profiles of 6 biological repeats are summarized in Fig. 1D and the percentage of cells with DNA > 4n are compared in Fig. 1E. Cells infected with Δ*E4orf1-3* Ad showed a consistent biphasic cell cycle profile with stark reduction in DNA > 4n relative to cells infected with Δ*E1B* Ad (Fig. 1, compare B row 3 to C row 1, and Fig. 1D and E). These results reveal a requirement for the E4orf1-3 gene-products in the Δ*E1B* Ad-induced DNA > 4n.

To determine which of the *E4orf1-3* gene-products were most responsible for the levels of DNA > 4n in the Δ*E1B* Ad infections, A549 cells were infected with Δ*E1B* Ad in which the E4orf3 gene was disrupted (30) (Δ*E4orf3* Ad, Fig. 1A), and the DNA cell cycle profile was evaluated. The Δ*E4orf3* Ad-infected cells showed a similar DNA cell cycle profile as the Δ*E4orf1-3* Ad-infected cells (Fig. 1C, compare row 1 to row 2, and Fig. 1E). As depicted in Fig. 1A, the Δ*E4orf3* Ad contains the Ad E4orf2 and E4orf1 genes that are deleted in the Δ*E4orf1-3* Ad. As of yet, no role has been ascribed to Ad E4orf2. Ad E4orf1 however, signals through the PI3 kinase (32, 33) and Rac1 (33) pathways. In other cell systems constitutively active PI3 kinase and Rac1 both induce DNA > 4n (34). Thus, in cells infected with Δ*E4orf3* Ad, E4orf1 may contribute to the DNA > 4n observed. To determine if E4orf1 supports DNA > 4n we created a Δ*E1B* Ad in which we deleted *E4orf1* (Δ*E4orf1 Ad*, Sangare et al., submitted) and compared the DNA cell cycle profile of the infected cells to that of the viruses in Fig. 1B and C. The Δ*E4orf1* Ad promoted higher fractions of cells with DNA > 4n than the Δ*E4orf3* Ad (Fig. 1C, compare row 2 to row 3) but not significantly so (Fig. 1E). The fraction of cells with DNA > 4n promoted by the Δ*E4orf1* Ad however, was significantly less than that observed in cells infected with the Δ*E1B* Ad (compare row 3 of Fig. 1B to row 3 of Fig. 1C; and Fig. 1E). We take these results to mean that in addition to E4orf3, E4orf1 also contributes to DNA > 4n observed in Δ*E1B* Ad-infected cells. However, *E4orf3*-deleted Ads (Δ*E4orf3* and Δ*E4orf1-3* Ad) over time consistently maintained lower fractions of cells with DNA > 4n compared to the *E4orf3*-containing Ads (Δ*E3*, Δ*E1B*, and Δ*E4orf1* Ad; Fig. 1). Thus, between Ad E4orf1, E4orf2 and E4orf3, the activity associated with Ad E4orf3 contributes the most to the DNA > 4n observed in Δ*E1B* Ad-infected cells. These experiments were also performed in HeLa cells with similar results (data not shown).

### Disruption of NBS1/MRN complex formation in Ad-infected cells.

**FIG 2:**
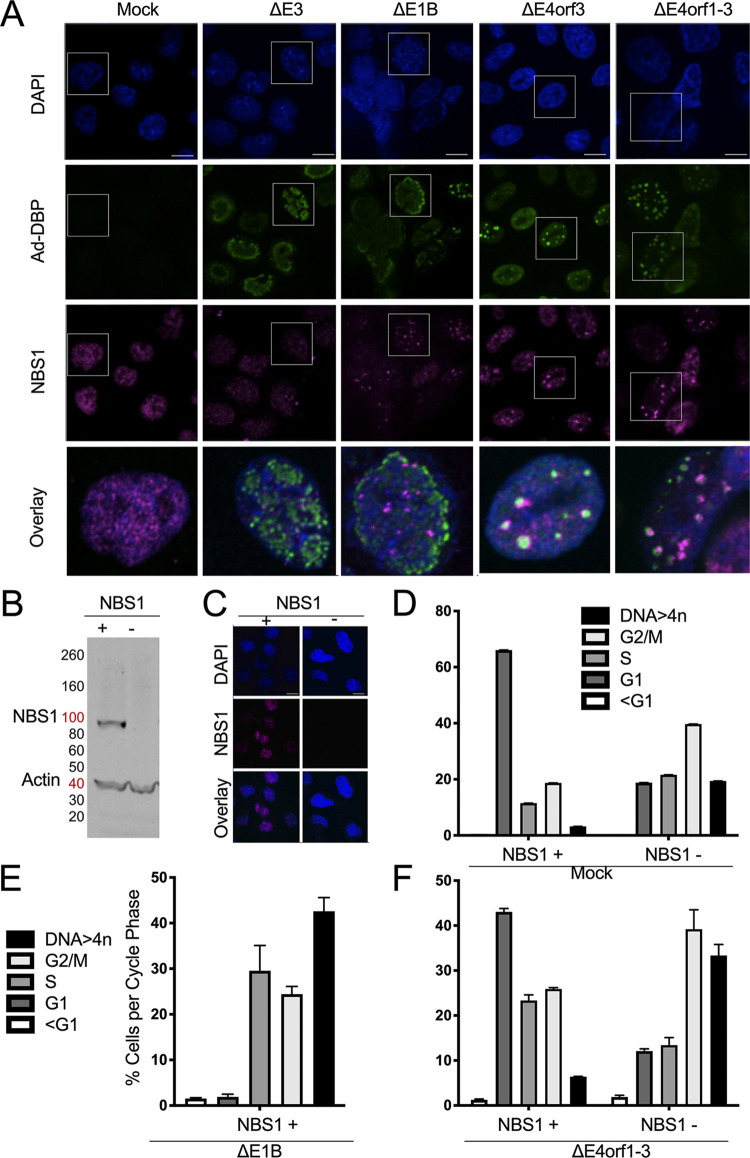
Disruption of NBS1 associates with DNA > 4n. (A) A549 cells were infected with the Ad listed at the top at an MOI of 50 for 48 h. In mock-infected cells, NBS1 (magenta) is evenly distributed throughout the nucleus. In cells infected with *E4orf3*-containing Ad (*ΔE3*, and *ΔE1B* Ad), NBS1 is redistributed into nuclear dots and/or tracks away from the viral replication centers marked by DBP (green). In cells infected with *E4orf3*-deleted Ad (*ΔE4orf3*, *and ΔE4orf1-3* Ad), NBS1 is localized to the viral replication centers. Cell nuclei were stained with DAPI (blue). (B–C) The status of NBS1 in NBS1^+^ and NBS1^−^ cells was verified by (B) Western blot and (C) immunofluorescence where NBS1 is in magenta and cell nuclei stained with DAPI are in blue. (D–F) For NBS1^+^ and NBS1^−^ cells, the % of cells per cycle phase are shown (mean ±SEM values of *n* = 4 independent experiments). (D) A representative example of the DNA cell cycle profiles of NBS1^+^ and NBS1^−^ cells. (E) Cell cycle distribution of NBS1^+^ cells infected with the *ΔE1B* Ad. (F) Cell cycle distribution of NBS1^+^ and NBS1^−^ cells mock-infected or infected with *ΔE4orf1-3* Ad. Scale bars in A and C indicate 10 μm.

What E4orf3 does in the infected cells to support DNA > 4n is not known. However, E4orf3 is widely reported to interfere with the ability of cells to transmit DDR signals by disrupting MRN complex formation (25–27). In some systems disruption of the MRN and/or DDR signals leads to DNA > 4n (28). The MRN complex consists of MRE11, RAD50 and NBS1. E4orf3 redistributes both MRE11 and NBS1 into nuclear tracts, effectively inactivating the MRN complex (25–27). To confirm that E4orf3 disrupts MRN complex formation in our experimental systems, we infected A549 cells and 48 hpi fixed, stained, and imaged them for the NBS1 (magenta) component of the MRN complex (Fig. 2A). For each infection a 3.2X-enlarged overlay is shown at the bottom. In the *E4orf3*-containing Ad infections (Δ*E3* and Δ*E1B*) NBS1 appeared in the nucleus, like the mock-infected cells, as indicated by DAPI in blue (Fig. 2A). However, in contrast to the mock-infected cells, in the Δ*E3* Ad infections NBS1 was reduced, and what remained, as in Δ*E1B* Ad infections were redistributed away from the Ad DNA binding protein, DBP in green (Fig. 2A). This indicates that here the MRN is inactivated (25). In the cells infected with the *E4orf3-*deleted Ads (Δ*E4orf3*, and Δ*E4orf1-3*) NBS1 was more abundant and to a large degree, appeared to overlap the Ad DNA binding protein (Fig. 2A) as expected of a functional MRN complex (25). Thus, in our hands, cells infected with *E4orf3*-containing Ad disrupted MRN complex formation, as was previously reported (25–27).

### Disruption of NBS1 is sufficient for DNA > 4n in ΔE1B Ad-infected cells.

To determine if disruption of NBS1 is sufficient for DNA > 4n, we obtained cells from a Nijmegen Breakage Syndrome (NBS) patient that lack the *NBS1* gene (NBS1^−^) and NBS cells that stably express wild-type *NBS1* (NBS1^+^) (35). In Fig. 2B we show the status of NBS1 in the NBS1^+^ and NBS1^−^ cells by Western blot and in Fig. 2C by immunofluorescence (IF). In Fig. 2D to F the DNA cell cycle profile of the NBS1^+^ and NBS1^−^ cells were assessed as in Fig. 1B and C and graphed as in Fig. 1D but without being stacked. In Fig. 2D, the cells that express NBS1 showed a rather normal DNA cell cycle profile with increased levels of G1 cells and low levels of DNA > 4n, similar to that observed in mock-infected A549 (Fig. 1B) and HeLa cells (data not shown). In contrast cells lacking NBS1 displayed an altered DNA cell cycle profile with a large fraction of cells in G2/M and some that escaped the cell cycle and accumulated DNA > 4n (Fig. 2D). A similar outcome was reported in B cells where NBS1 was disrupted (4). These results suggest that NBS1 and/or the MRN complex control progression through the cell cycle.

Because Δ*E1B* Ad promotes the largest fraction of cells with DNA > 4n (Fig. 1) we infected NBS1+ cells with this Ad as a positive control (Fig. 2E). In Fig. 2E, compared to the mock (Fig. 2D [black bar]), the Δ*E1B* Ad promoted a high percentage of NBS1^+^ cells with DNA > 4n. Correspondingly, infection of the NBS1+ cells with the Δ*E4orf1-3* Ad that is unable to disrupt NBS1 (Fig. 2A), led only to a marginal increase in cells with DNA > 4n (Fig. 2F). In contrast, in NBS1^−^ cells the same Δ*E4orf1-3* Ad promoted DNA > 4n (Fig. 2F) reminiscent of the Δ*E1B* Ad (Fig. 2E) that has the ability to disrupt NBS1 (Fig. 2A). Although here we did not formally prove that E4orf3 can independently induce DNA > 4n, we demonstrated that the activity for which E4orf3 is perhaps best known, disruption of MRN complex formation, is sufficient for elicitation of enhanced levels of DNA > 4n in ΔE1B Ad-infected cells.

### Disruption of NBS1 associates with enhanced levels of nuclear NF-κB in Ad-infected cells.

**FIG 3:**
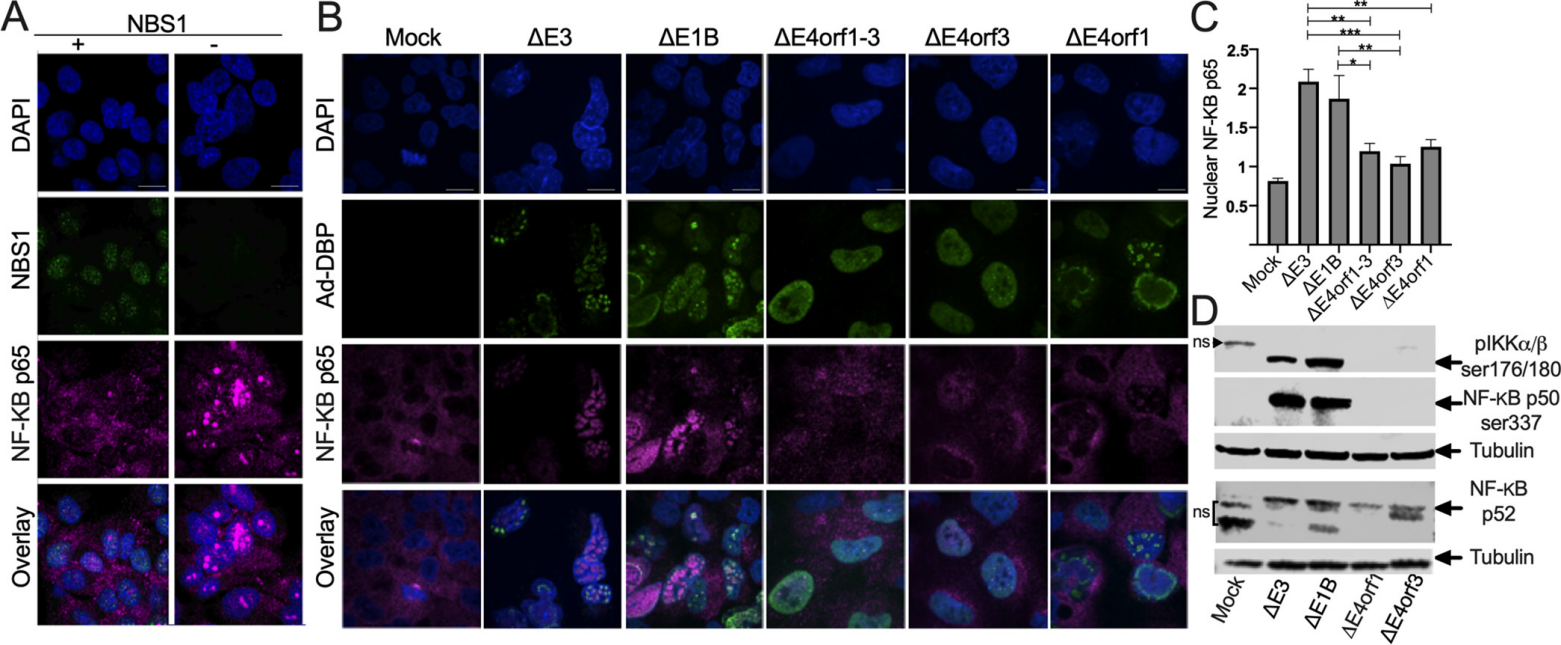
Ad infections lead to canonical and noncanonical activation of NF-κB. (A) The status of NF-κB p65 was evaluated by IF in NBS1^+^ and NBS1^−^ cells where NBS1 is in green, NF-κB p65 in magenta and cell nuclei stained with DAPI are in blue. (B-D) A549 cells were infected with the listed Ad at an MOI of 50 for 48 h. (B) Immunofluorescent staining showing Ad DNA binding protein (DBP) in green and NF-κB p65 (magenta). Cell nuclei were stained with DAPI (blue). Scale bars in A and B indicate 10 μm. (C) Nuclear NF-κB p65 was quantified using ImageJ software. Graph illustrates mean ±SEM values of n = 3 independent experiments. *P* values were obtained using one-way ANOVA where *, *P* < 0.05; **, *P* < 0.01; ***, *P* < 0.001. Western blot images of (D) phospho-IKKα/β (Ser176/180), phospho-p50 (Ser337) and p52 are shown with nonspecific bands labeled as ns. (See also Fig. S1 for entire gel).

How cells survive with DNA > 4n or for that matter with disruption of NBS1 and/or the MRN complex is not clear. However, NF-κB has been linked to DNA damage (36–38) that is mediated by NBS1 and the MRN complex. To learn if and how NF-κB is linked to NBS1, we compared the level of nuclear NF-κB in NBS1^+^ and NBS1^−^ cells. In Fig. 3A a representative example of immunostained NBS1^+^ and NBS1^−^ cells is shown. The levels of nuclear NF-κB were calculated by first identifying the DAPI stained nuclei regions of interest (ROI) and using ImageJ/Fiji software to quantify the levels of the NF-κB p65 signals within the applied ROI. By these measures, the nuclear levels of NF-κB p65 were significantly higher (*P* < 0.01) in the NBS1^−^ cells compared to the NSB1^+^ cells (data not shown).

**FIG 4:**
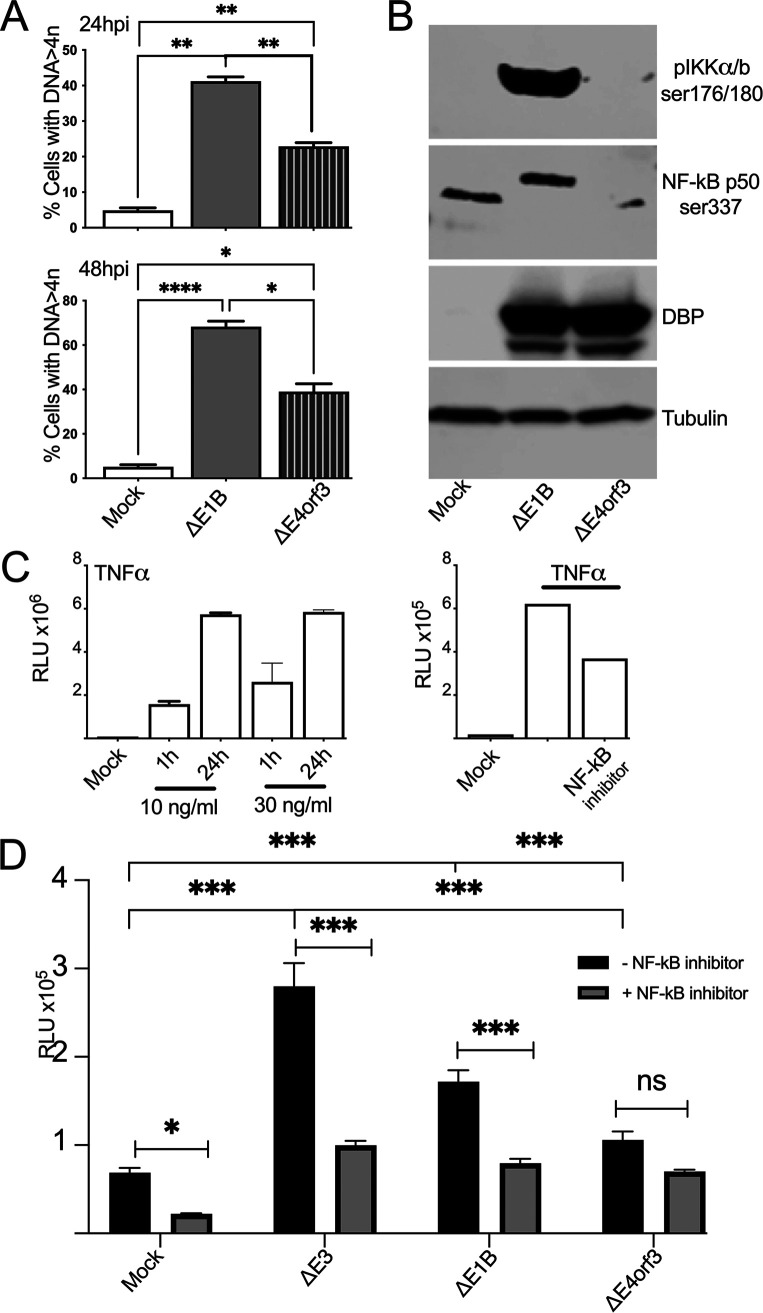
Ad-infections promote NF-κB transcriptional activity. HCT116-luc cells were not infected (mock) or infected with the indicated virus at an MOI of 50 for 24 or 48 h. (A) The percentage of cells with DNA > 4n with the mean ±SEM is shown for *n* = 6 independent experiments. (B) Western blot images of the static levels of phospho-IKKα/β (Ser176/180), phospho-p50 (Ser337), the infection control Ad DNA binding protein (DBP), and the loading control tubulin are shown. See also Fig. S2 for entire gel. (C) The relative luciferase activities of TNFα treated HCT116-luc cells (left bar graft) and TNFα treated HCT116-luc cells additionally treated with the NF-κB specific inhibitor SC7541 (right bar graph) are shown. (D) The relative luciferase activities of Ad-infected HCT116-luc cells (black bars) or Ad-infected HCT116-luc cells treated with the specific NF-κB inhibitor SC7541 (gray bars) 48 hpi are shown. Graph illustrates mean ±SEM values of *n* = 3 independent experiments. *P values* were obtained using two-way ANOVA where *, *P* < 0.05; ***, *P* < 0.001.

To discover the activation status of NF-κB during Ad infection, A549 cells were infected with Δ*E3*, Δ*E1B*, Δ*E4orf1-3*, Δ*E4orf3* or the Δ*E4orf1* Ad and stained as described in the Methods section (Fig. 3B). In these experiments, cells infected with Δ*E3* or with Δ*E1B* Ad exhibited significantly higher levels of nuclear NF-κB p65 than cells infected with Δ*E4orf1-3*, Δ*E4orf3*, or Δ*E4orf1* Ad (Fig. 3B and C). In the canonical NF-κB pathway, IKKα and IKKβ serve as the catalytic subunits for the IκB kinase (IKK) complex that phosphorylates IκB (39). Upon phosphorylation of IKKα on Ser180 and IKKβ on Ser176 (40–43) the complex phosphorylates IκB leading to its ubiquitination and proteasome-degradation (44) that results in the release and nuclear translocation of NF-κB. In Fig. 3D we show a Western blot image of phospho-IKKα/β (ser176/180) in mock-infected cells and cells infected with Δ*E3*, Δ*E1B*, Δ*E4orf3* and the Δ*E4orf1* Ad. Consistent with Fig. 3B and C, here cells infected with Δ*E3*, and Δ*E1B* Ad have higher levels of phospho-IKKα/β (ser176/180) than cells infected with Δ*E4orf3* and the Δ*E4orf1* Ad (Fig. 3D). NF-κB p50 forms homodimers or heterodimerizes with p65 to form the functional NF-κB. Phosphorylation of p50 at Ser337 is required for DNA binding (45). In the second row of Fig. 3D we show phospho-p50 (ser337) in the various Ad-infected cells. Cells infected with Δ*E3* and Δ*E1B* Ad exhibited higher levels of phospho-p50 (ser337) than cells infected with Δ*E4orf3* and the Δ*E4orf1* Ad. Another member of the NF-κB family, p52 is held in the cytoplasm as a p100 precursor protein. The processing of p100 to p52 is activated through the noncanonical pathway (46). In the bottom part of Fig. 3D, we show a Western blot using an antibody that recognizes the p52 subunit. Cells infected with Δ*E3* and Δ*E1B* Ad exhibited higher levels of p52 than cells infected with Δ*E4orf3* and the Δ*E4orf1* Ad. The entire gels for the blots shown in Fig. 4D are shown in Fig. S1. The bands labeled ‘ns’ may be nonspecific as they do not appear in other cell lines or are also in the mock. Nonetheless, taken together the data suggest that in cells infected with *E4orf3*-containing Ad (Δ*E3* and Δ*E1B* Ad) that we showed in Fig. 2A disrupts NBS1, both the canonical and noncanonical NF-κB pathways are activated.

### NF-κB is transcriptionally active in Ad-infected cells.

NF-κB is a transcription factor that supports the expression of hundreds of target genes (47). To determine if the NF-κB that translocates to the nucleus after the disruption of NBS1 and/or the MRN complex participates in transcription, HCT116 NF-κB luciferase reporter cells (HCT116-luc) (48) were mock-infected or infected with *ΔE1B* Ad or *ΔE4orf3* Ad and levels of DNA > 4n were assessed. Similar to Fig. 1 and 2 above, HCT116-luc cells infected with the Δ*E1B* Ad supported a higher percentage of cells with DNA > 4n than HCT116-luc cells infected with the Δ*E4orf3* Ad at both 24 and 48 hpi (Fig. 4A). In HTC116-luc cells infected with the Δ*E1B* Ad, the static levels of phospho-IKKα/β (ser176/180) and phospho-p50 (ser337) were elevated compared to cells infected with the Δ*E4orf3* Ad (Fig. 4B, and Fig. S2 for the entire gels). The specific bands in Fig. 4B are similar to those observed in the A549 cells in Fig. 3D. In Fig. 4C, and elsewhere (48) treating the HCT116-luc cells with TNFα, known to induce NF-κB activation (49), dramatically increased the luciferase activity (here measured in RLU). HCT116-luc cells infected with the *E4orf3*-containing Δ*E3* and Δ*E1B* Ad exhibited significantly higher levels of luciferase activity compared to those infected with the Δ*E4orf3* Ad (Fig. 4D compare black bars). To further validate our system, we infected the HCT116-luc cells and 4 h later treated them with an NF-κB-specific inhibitor, SC75741. In Fig. 4C (the right bar graph), we show that the NF-κB-specific inhibitor is able to diminish the induction of NF-κB in cells treated with TNFα. In a similar fashion, mock-infected and Ad-infected HCT116-luc cells that were treated with the NF-κB inhibitor showed reduced levels of luciferase and, by extension, NF-κB transcriptional activity (Fig. 4D). These results support the data shown in Fig. 3 and allow for the conclusion that the NF-κB that translocates to the nucleus after disruption of NBS1 is transcriptionally active.

### Pharmacological inhibition of NF-κB reduces DNA > 4n in ΔE1B Ad-infected cells.

**FIG 5:**
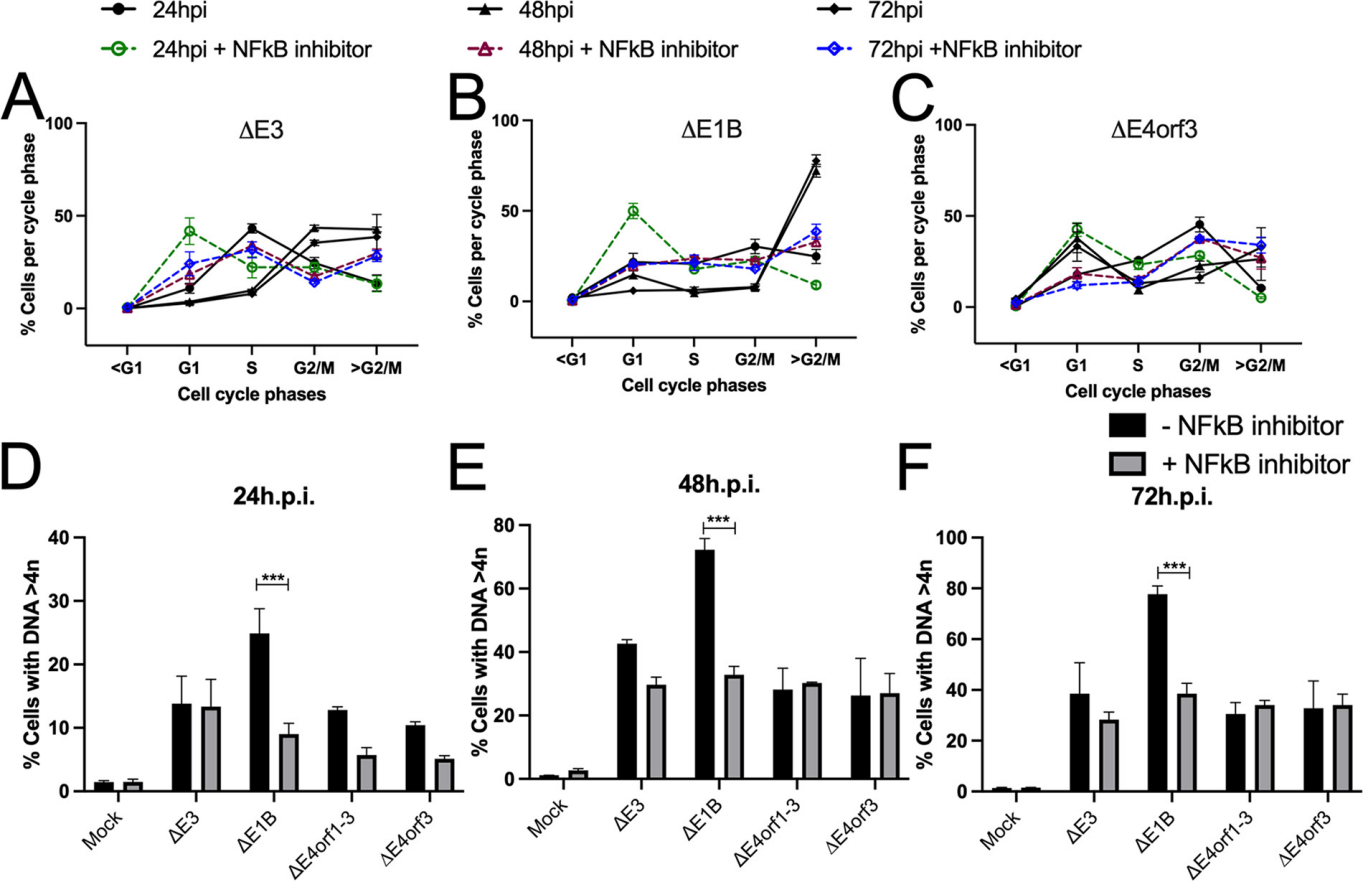
Inhibition of NF-κB reduces DNA > 4n in Ad-infected cells. The DNA cell cycle profiles of A549 cells infected with (A) *ΔE3* Ad, (B) *ΔE1B* Ad, and (C) *ΔE4orf1-3* Ad. Four hours after infection, the cells were treated with 5 μM of NF-κB inhibitor, SC75741. The percentages of cells with DNA > 4n were quantified (D) at 24 hpi, E) 48 hpi, and F) 72 hpi. Graphs D–F illustrate mean ±SEM values of *n* = 4 independent experiments. *P values* were obtained using two-way ANOVA where ***, *P* < 0.001.

To assess the impact of NF-κB on levels of cellular DNA > 4n, A549 cells were mock infected or infected with the indicated Ad and treated 4 h later with or without the NF-κB-specific inhibitor SC7574. The DNA cell cycle profiles were assessed by flow cytometry as before. In Fig. 5A to C, DNA cell cycle profiles of Ad-infected A549 cells treated with (colored lines and bars) or without (black lines and bars) SC75741 are shown. In Fig. 5D to F we compare the fraction of cells with DNA > 4n. From these results it appears that inhibiting NF-κB reduces the levels of DNA > 4n to a similar extent as deleting *E4orf3* from the Δ*E1B* Ad. Thus, the enhanced levels and activity of nuclear NF-κB (Fig. 3 and 4) associates with DNA > 4n (Fig. 5) in Δ*E1B* Ad-infected cells.

### ATM mediates NF-κB activation and DNA > 4n in ΔE1B Ad-infected cells.

**FIG 6:**
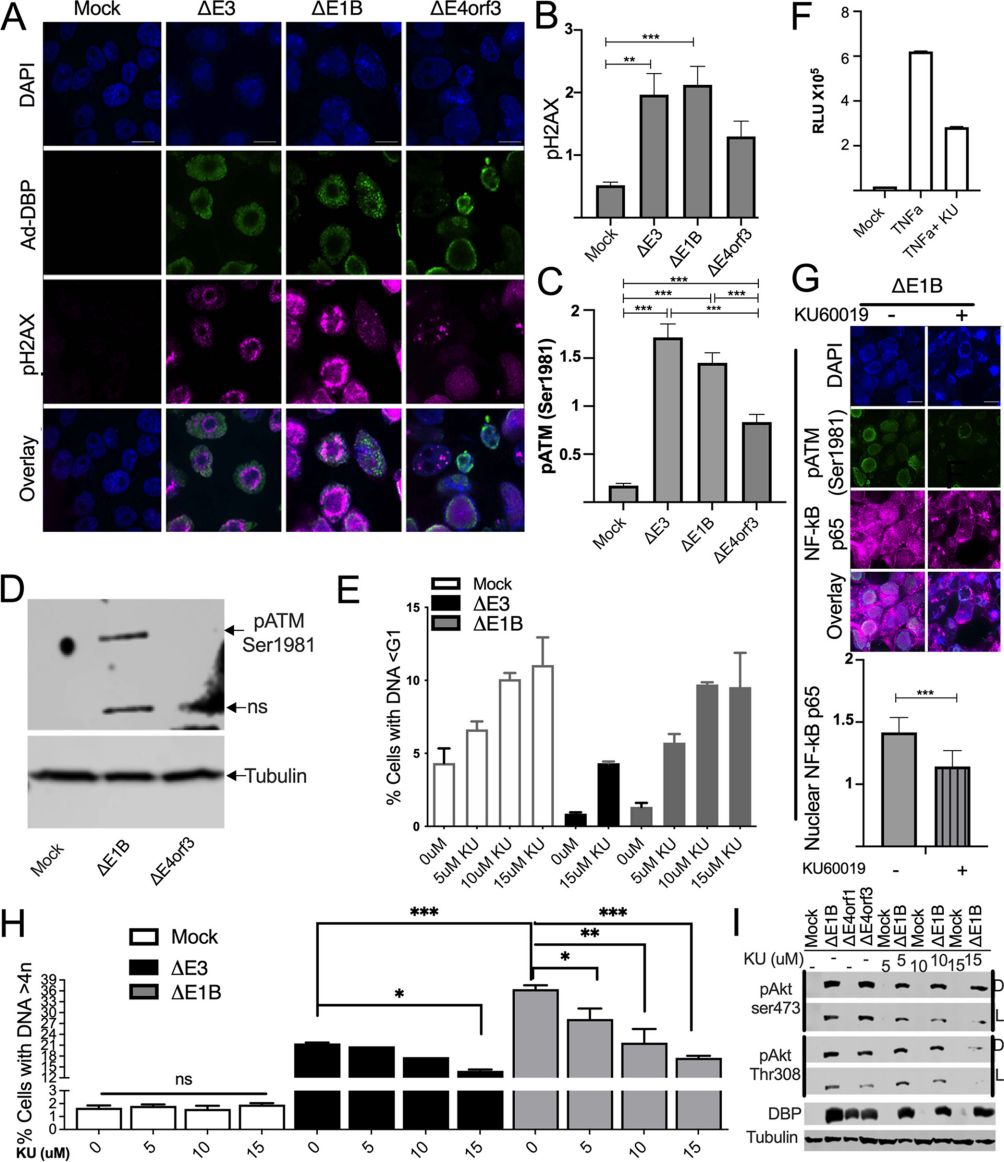
Inhibition of ATM kinase activity reduces DNA > 4n and NF-κB activation in Ad-infected cells. HeLa cells were infected with the indicated Ad at an MOI of 50 for 48 h. (A) Immunofluorescent staining showing pH2AX ser139 (magenta), Ad DNA binding protein (DBP) (green) and cell nuclei stained with DAPI (blue). Fluorescent intensity of (B) pH2AX ser139 and (C) pATM ser1981 were quantified using ImageJ. (D) Western blot image of the static levels of phospho-ATM ser1981 are shown in HTC116-luc cells. See also Fig. S3 for entire gel. (E) The percentages of cells with DNA content less than G1 (DNA<G1) for mock or cells infected with *ΔE3*, or *ΔE1B* Ad and treated with ATM inhibitor, KU60019 are shown for *n* = 6 independent experiments. (F) The relative luciferase activities of TNFα treated HCT116-luc cells that were additionally treated with or without the ATM kinase inhibitor KU60019 are shown. (G) Immunofluorescent staining of A549 cells infected with *ΔE1B* Ad that were treated with or without ATM inhibitor. pATM ser1981 (green), NF-κB p65 (magenta), and cell nuclei stained with DAPI (blue). Scale bars in A and G indicate 10 μm. Nuclear NF-κB p65 was quantified using ImageJ software. (H) The percentages of cells with DNA > 4n among cells that were not infected (mock) or infected with *ΔE3*, or *ΔE1B* Ad and treated with ATM inhibitor, KU60019. The percentages of cells with DNA > 4n were quantified 48 hpi. Graph illustrates mean ±SEM values of *n* = 4–6 independent experiments. *P values* were obtained using one-way ANOVA where **, *P* < 0.01; ***, *P* < 0.001. (I) HeLa cells were infected with the indicated Ad at an MOI of 50 and 4 hpi treated with or without ATM inhibitor, KU60019 at increasing concentrations. Western blot image of the static levels of phospho-Akt (Ser473) and Thr308 are shown 48 hpi. D = long exposure; L = short exposure. See also Fig. S4 for entire gel.

We next explored some of the possible means by which NF-κB may be activated in Ad-infected cells. Of these, ATM either directly or indirectly phosphorylates NF-κB (36, 50–52) and is required for NF-κB activation after DNA damage (36, 37). After DSB the MRN complex sequesters the major kinases ATM and ATR (6–8). ATM is activated and phosphorylates a series of DDR-related substrates, including two major transcription factors, p53 and NF-κB (53). Among the p53-independent responses, ATM interacts with the NF-κB essential modulator (NEMO or IKKγ) (39) that is credited with facilitating the proteasomal degradation of phospho-IκB (52, 54). To investigate the possible involvement of ATM in the activation of NF-κB and the induced DNA > 4n we first sought to evaluate the status of phosphorylated ATM in Ad-infected cells. Because the Ad DNA binding protein (DBP) antibody was made in the same species as our ATM antibody we could not evaluate the two in the same reaction and so do not show those images. Instead in Fig. 6A we show IF images of Ad DBP in green and higher levels of phospho-H2AX (magenta) in the nucleus (DAPI: blue) of the Ad-infected HeLa cells. H2AX is a downstream target of ATM (55). These experiments were repeated (*n* = 6) and the nuclear levels of phospho-H2AX were quantified and plotted, revealing smaller amounts of phosphorylated H2AX in Δ*E4orf3* Ad-infected HeLa cells (Fig. 6B). By the same measure levels of phosphorylated ATM were even more reduced (Fig. 6C). To further confirm the levels of phosphorylated ATM, a Western blot was performed on the HCT116-luc cell samples used in Fig. 4 (Fig. 6D, and Fig. S3 for entire gel). While more exaggerated, the Western blot recapitulated the IF results in Fig. 6C showing higher levels of phosphorylated ATM in cells infected with the Δ*E1B* Ad compared to the Δ*E4orf3* Ad-infected cells (Fig. 6D). Because the Δ*E4orf3* Ad supports significantly lower levels of cells with DNA > 4n than the Δ*E1B* Ad (Fig. 1 and 4, and 5), it appears that here too, ATM may serve as a cornerstone to bridge the nuclear DNA damage response to activation of NF-κB (56).

To determine if ATM played a role in the induced DNA > 4n and/or the elevated levels of the accompanying nuclear NF-κB, HeLa cells were infected with Δ*E3* or Δ*E1B* Ad and 4 h later were treated with KU60019 that has been reported to block ATM activity (57–59). A study using a similar ATM inhibitor in Ad-infected cells has been reported (60). The DNA cell cycle profiles were assessed by flow cytometry as above. In these analyses, cells with DNA content less than the G1 phase of the cell cycle (DNA<G1) represent dead or dying cells with fragmented DNA (61). At the highest concentration used, 15 μM, the ATM inhibitor KU60019 increased the fraction of cells with fragmented DNA (DNA<G1) to about 10% in mock- and Δ*E1B* Ad-infected cells, but only to about 5% in Δ*E3* Ad-infected cells (Fig. 6E). This revealed a major difference in the ability of cells infected with Δ*E3* Ad to survive genotoxic stress compared to cells infected with Δ*E1B* Ad. Even though the E4 products, some of which are considered redundant to the E1B55K/E4orf6 complex with regard to disruption of MRN complex formation (25), are present in the Δ*E1B* Ad (see list in Fig. 1A), the fraction of cells with fragmented DNA was increased above that induced by treatment of the Δ*E3* Ad-infected cells with the ATM inhibitor (Fig. 6E). In HCT116-luc cells treated with TNF the ATM inhibitor KU60019 decreased induction of NF-κB (Fig. 6F). IF images in Fig. 6G show levels of NF-κB p65 (magenta), and phospho-ATM (green) in the nucleus (DAPI: blue) of Δ*E1B* Ad-infected HeLa cells treated with or without the ATM inhibitor KU60019. In these cells, the nuclear levels of NF-κB were significantly reduced (*P* < 0.001) after treatment with the ATM inhibitor KU60019 (Fig. 6G). These results suggest that in Δ*E1B* Ad-infected cells, preventing ATM activity reduces the level of nuclear NF-κB (Fig. 6G) and leads to cell death (Fig. 6E).

To assess the impact of ATM activity on the Ad-induced DNA > 4n, HeLa cells were infected with Δ*E3* or Δ*E1B* Ad and treated with or without the ATM kinase inhibitor KU60019. In Fig. 6H we show that over a range of concentrations the ATM inhibitor KU60019 reduced the levels of DNA > 4n in Ad-infected cells. As in Fig. 6E, the Δ*E1B* Ad displayed greater sensitivity to the ATM inhibitor KU60019, showing significant reduction in DNA > 4n even at the lowest concentration.

**FIG 7:**
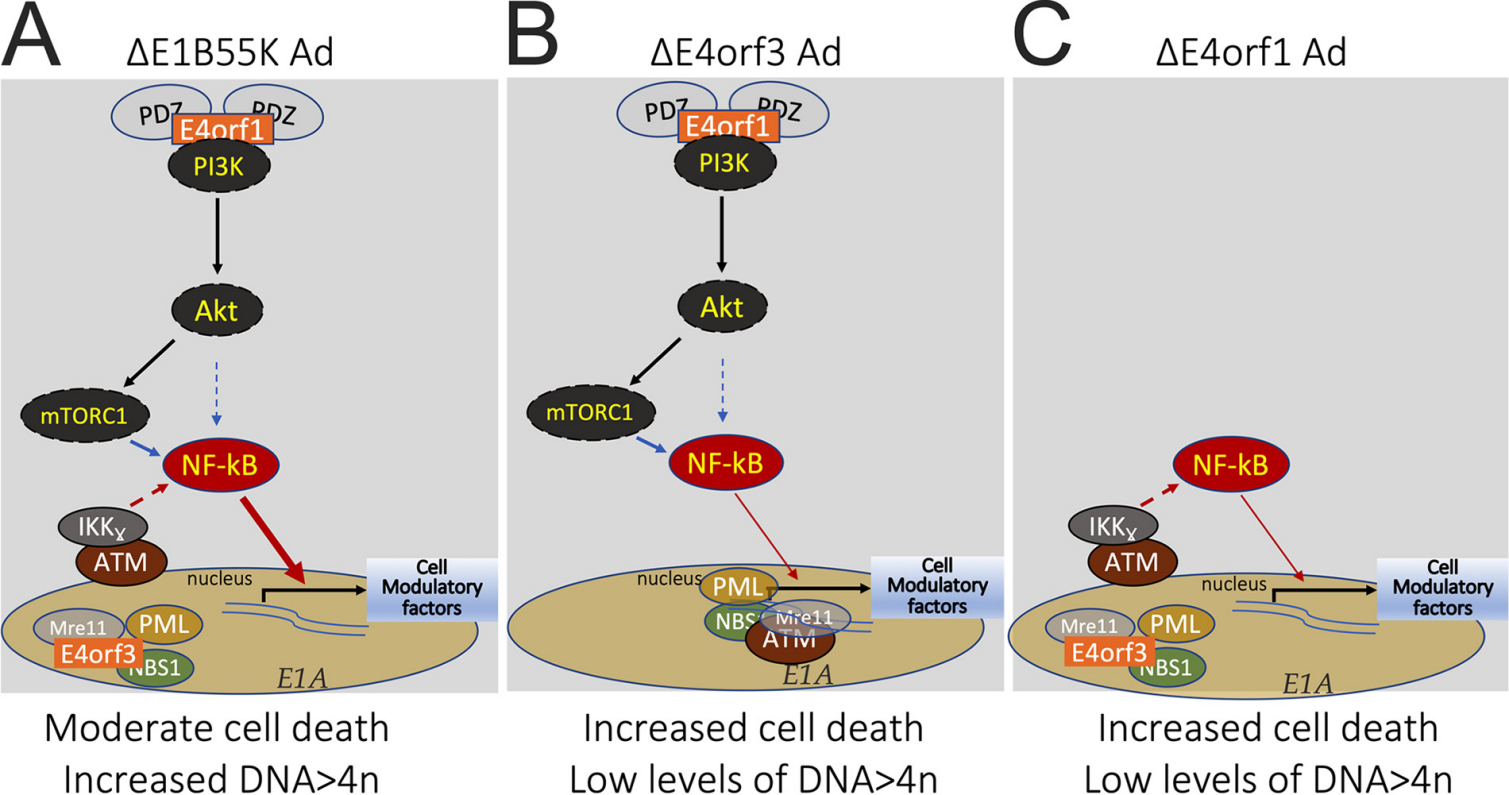
Simplified illustration of events that support DNA > 4n in ΔE1B Ad. In wild type Ad E1A induces DNA damage and/or promotes DDR signals that can trigger cell death. The Ad E1B55K and E4orf6 form a complex that promotes the degradation of several DDR proteins, effectively nullifying the E1A-induced DDR signals. (A) In the *ΔE1B* Ad-infected cells, E4orf3 prevents the E1A-induced DDR signals by sequestering NBS1 and Mre11 in the nucleus. Since the main role of NBS1 is to guide ATM and ATR to sites of DNA damage, in cells where NBS1 is disrupted ATM and ATR are free to participate in other activities some of which promote the phosphorylation of NF-κB. By itself the activity of E4orf3 may not be sufficient to prevent the E1A-induced DDR signals. However, E4orf1 through PDZ interactions stimulates the PI3-kinase (PI3K)/Akt/mTORC1 pathway that also promotes the phosphorylation of NF-κB. The activity of both E4orf1 and E4orf3 reprograms NF-κB thus allowing the Ad infected cells to survive with enhanced levels of DNA > 4n. (B) In the *ΔE4orf3* Ad, while E4orf1 continues to signal through the PI3K/Akt/mTORC1 pathway this by itself is not sufficient to prevent the E1A-induced DDR signals. In these cells, perhaps due to a functional MRN, cell death is enhanced while DNA > 4n is limited. (C) In the *ΔE4orf1* Ad-infected cells, while E4orf3 continues to disrupt MRN complex formation this by itself is not sufficient to prevent the E1A-induced DDR signals. Here, too, cell death is enhanced and DNA > 4n while more than in *ΔE4orf1* Ad-infected cells remains reduced relative to *ΔE1B* Ad-infected cells.

Recently it was suggested that ATM forms a scaffold upon which Akt sits (62). Thus, agents that destabilize ATM attenuate Akt phosphorylation and induce cell death (62, 63). For that reason, we sought to determine if inhibition of ATM activity by the inhibitor KU60019 reduces Akt phosphorylation in Ad-infected cells. HeLa cells were infected with the Δ*E1B* Ad for 4 h before they were incubated with or without the ATM inhibitor KU60019 (Fig. 6I, and Fig. S4 for entire gel). Relative to mock-infected cells, infection with the Δ*E1B* Ad induced elevated levels of Akt phosphorylated at Ser473 and Thr308 (Fig. 6I, Sangare et al., submitted, and [33]). This is additionally illustrated in Fig. 7A where we outline the events that support DNA > 4n in *ΔE1B* Ad-infected cells. The deletion of E4orf1 in Δ*E4orf1* Ad returned the phosphorylation of Akt back to the level of mock-infected cells (Fig. 6I, 7C). The Δ*E4orf3* Ad contains E4orf1 (Fig. 1A, 7B). Thus, in infections with Δ*E4orf3* Ad, E4orf1 also promotes the phosphorylation of Akt at Ser473 and Thr308 (Fig. 6I, 7B). As in noninfected cells (62), and shown here in Δ*E1B* Ad-infected cells, the phosphorylation of Akt on both Ser473 and Thr308 was reduced by increasing concentrations of KU60019 (Fig. 6I). This does not affect the conclusions drawn from Fig. 6A to H because as cells die (as with the ATM inhibitor KU60019, Fig. 6D and [62]) pathways that support survival are turned off. In fact, Akt deactivation occurs in multiple models of cell death (64). Note specifically however, at lower concentrations of KU60019 where Akt phosphorylation is only marginally affected (Fig. 6I), the percentage of cells with DNA > 4n is significantly reduced (Fig. 6G). Thus, taken together, these results suggest that in *E4orf3*-containing Ad-infected cells, ATM is needed for survival and for the NF-κB activity that facilitates DNA > 4n.

## DISCUSSION

Up until a specific point in early development disruption of NBS1 is embryonically lethal (65). Patients surviving with disruption of NBS1 exhibit Nijmegen breakage syndrome (NBS) and other diseases characterized by genomic instability (66–69). One question that has remained is how cells with disrupted NBS1 and/or MRN complex formation survive? This inactivation of genes that regulate the response to DNA damage is commonly found in human cancer (70, 71). Among other things, NBS1 recruits ATM and ATR to sites of DNA damage (38) where they phosphorylate members of the MRN complex and thereafter participate in DDR-related activities (72, 73) including the activation of cellular checkpoints that control cell cycle progression, repair of DNA breaks, and cell death (74–76). Here, in NBS1^−^disrupted cells (Fig. 2), we showed that ATM is important for cell survival, in part due to its relationship to NF-κB as we showed in Fig. 6 and Akt as we showed in Fig. 6I and illustrated in Fig. 7. Pharmacological inhibition of ATM activity reduced levels of nuclear NF-κB and enhanced levels of cells with fragmented DNA (DNA<G1 in Fig. 6). These results are consistent with the idea that ATM is indispensable for NF-κB activation (36, 37) and cell survival (Fig. 6). ATM has been reported to physically interact with NF-κB p65 and p50 (53) and in complex with IKKγ/NEMO promotes the phosphorylation of IKKα/β. While we did not confirm these physical interactions, they may explain the phosphorylation of IKKα/β we observed in Fig. 3D, and 4B. In our model (Fig. 7) the newly freed NF-κB translocates into the nucleus where it engages in transcriptional activity (Fig. 4) that supports cell survival that we showed in Fig. 6E. Thus, in cells with disruption in NBS1 and/or MRN complex formation, ATM supports NF-κB that allows the cells to survive with DNA > 4n.

In infections with the wild type Ad, levels of DNA > 4n remain unchanged independent of E4orf1, E4orf1-2, E4orf1-3, or E4orf1-4 (29). This occurs in spite of constitutively active PI3-kinase (34) as well as disruption of MRN complex formation (4), which are known to promote DNA > 4n. One possible explanation is that the roles and functions carried out by E1B55K (most likely in complex with E4orf6) in wild type Ad-infected cells, mask the E4 contribution to DNA > 4n. However, deletion of E1B55K resulted in higher fractions of cells with DNA > 4n than wild type Ad (Fig. 1, 5 and 6 and [30]) where deletion of E4orf1 to E4orf4 did not (29). Therefore, the actions carried out by the E1B55K/E4orf6 complex (with regard to DNA > 4n) do not merely duplicate those carried out by the other E4 proteins. For one, as shown in Fig. 6E levels of fragmented DNA were much lower in Δ*E3* Ad-infected cells treated with KU than in similarly treated Δ*E1B* Ad-infected cells. Taken to its logical end, the Ad5 E1B55K and E4orf6 complex seems more adept at regulating cell death/survival than either E4orf1, E4orf2, E4orf3 and E4orf4 combined. This as we postulated above, may explain why even though E4orf1 and E4orf3 are in the wild type Ad, and the individual pathways they mediate are active as we showed in Fig. 2, 3, 6 and 7, inhibition of these pathways only affect DNA > 4n at the higher inhibitor concentrations (Fig. 6H).

E1A has been reported to inhibit NF-κB activities (77). E1A and E4orf3 work together to, among other things, transform cells (21) and fine-tune the viral transcriptional program (78). During an Ad infection E1A is expressed early and in most cases, is severely reduced by 48 hpi (29). As we show in Fig. 1, infection with Ad results in a massive increase in DNA > 4n that can be observed as early as 24 hpi and continues to increase thereafter. Thus, if E1A acts to suppress NF-κB, that repression may be lost at the times when the increased DNA content is observed. A recent study showed that E1A by itself is sufficient to drive DNA > 4n in transfected cells (16). This, as we noted earlier, is accomplished through interaction with p300/CBP, TRRAP/p400 multiprotein complex and the retinoblastoma (pRb) family of proteins (12–15). Interestingly, some of these are transcriptional coactivators of NF-κB (79). Since E4orf3 is reported to cooperate with E1A to transform cells (21) and data shown here (Fig. 6) and by others elsewhere (80) reveal that an E4orf3 activity supports survival pathways, then it is possible that in E1A-transformed cells, E4orf3 may act similarly to support survival.

Viral activation of NF-κB is not unique. In fact, disparate virus families activate NF-κB to promote viral replication, to prevent cell death, and/or to mediate immune responses (81). In Fig. 3 and 4, levels of nuclear/transcriptionally activated NF-κB in cells infected with either Δ*E3* or Δ*E1B* Ad are similar. Yet wild type Ad produces much more late viral protein and progeny than Δ*E1B* Ad (33). Also, even though cells infected with Δ*E1B* Ad have higher levels of nuclear/activated NF-κB than those infected with Δ*E4orf1* Ad (Fig. 3), the cells infected with Δ*E1B* Ad produce significantly less late viral proteins and progeny than cells infected with Δ*E4orf1* Ad (Sangare et al., submitted and [33]). Thus, it is doubtful that the enhanced nuclear levels of NF-κB act to promote and/or sustain Ad proteins and/or progeny yield.

An immune modulatory role of NF-κB seems plausible, although we did not test that here. E4orf3 is known to inactivate viral-induced type 1 interferon responses (82) and in a recently submitted article we show that Δ*E4orf1* Ad induces higher levels of cytokine-related genes in cells and higher levels of transgene-specific immune responses in nonhuman primates than Δ*E1B* Ad (Sangare et al., submitted). Thus, both Ad E4orf1 and E4orf3, even while they support NF-κB, negatively impact immune responses. Therefore, if the enhanced nuclear levels of NF-κB in the Ad-infected cells are induced to notify the immune system of the ongoing infection, this may be effectively limited by E4orf1 (Sangare et al., submitted) and E4orf3 (82).

We conclude that since E4orf3 is a viral oncogene (21, 22), in malignancies characterized by the disruption of NBS1 or MRN complex formation, blocking signals mediated by ATM, or NF-κB may constitute treatment options.

## MATERIALS AND METHODS

### Contact for reagent and resource sharing.

Further information and requests for resources and reagents should be directed to michael.thomas1@howard.edu.

### Ethics statement.

All experiments were approved by the Institutional Biosafety Committee (IBC) at Howard University.

### Antibodies.

**(i) For western.** NBS1 2 μg/ml, pIKKα/β(ser176/180) 1:1000, β-Tubulin 1:5000, NF-κB p100/52 1:1000, NF-κB p105/50 1:1000, p53 (DO1) 1 μg/ml, Actin 1:5000, Ad type 5 1:2000, Mad2 1:1000, pH2AX (ser139) 1:1000, DBP 1:2000, ATM 1:1000, NBS1 1:1000, MRE11 1:1000, Akt Ser473 1: 1:1000, Akt Thr308 1: 1:1000.

**(ii) For immunofluorescence.** Ad-DBP 1:1000, NBS1 2 μg/ml, NF-κB p65 1 μg/ml, NF-κB p100/52 1:500, NF-κB p105/50 1:500, pATM (ser1981) 5 μg/ml, pH2AX (ser139) 1:500.

### Experimental models.

**(i) Viruses.** The mutant virus *dl*1520 listed here as *Δ*E1B contains an 827-bp deletion in the region encoding the 55 kDa protein (31). The *Δ*E4orf3 Ad, *dl*3112 (83) contains the E1B55K-deletion from *dl*1520 and the E4orf3 inactivation-mutation from *dl*341 (84). The *dl*1018 Ad listed here as *Δ*E4orf1-2 contains deletions in the regions encoding E1B55K and E4orf1-2 (85). The *dl*1016 Ad listed here as *Δ*E4orf1-3 contains deletions in the regions encoding E1B55K and E4orf1-3 (85). The phenotypic wild type *ΔE3* Ad, was described elsewhere (29). The ΔE1B55K and E4orf1-deleted Ad, listed here as *Δ*E4orf1 was created by recombining SpeI digested *dl*1520 DNA with BamHI digested pBRAd5hr*ΔE3* plasmid in which we deleted the coding regions for E4orf1 as described elsewhere (29). The resulting viral DNAs were screened by PCR for the presence or absence of *E1B55K* and E4orf1. The viruses were purified, and concentrations determined as described (33) by plaque assays.

### (ii) *In vitro* studies: cell culture.

We used the human cervical carcinoma-derived HeLa cell line (ATCC CCL-2) because most of what is known about Ad is in the context of this cell line. We used the human lung A549 cell line (ATCC CCL-185) as Ad is known to target the upper respiratory air way. The NBS1 negative cell line (ILB-1) and ILB-1 cells expressing wild-type NBS1 (NBS1) were obtained from Dr. Patrick Concannon (University of Florida). HCT116 cells that contain NF-κB luciferase reporter (HCT116-luc) were obtained from Dr. Temegsen Samuel (Tuskegee University). Cells were cultured in Dulbecco's Modified Eagle *Medium* supplemented with 10% Fetal Bovine Serum (FBS), 100 unit/ml Penicillin and 100 mg/ml Streptomycin. NBS1 and ILB-1 cells were maintained in complete media as described above and 500 μg/ml G418. HCT116 cells were maintained in complete media and 1 μg/ml puromycin. The cells were incubated at 37°C in humidified air under 5% C0_2_. PI3-kinase inhibitor LY294002, MAPK/ERK1/2 inhibitor U0126 and PD98059, NF-κB inhibitor SC75741, and ATM inhibitor KU-60019 were obtained from Selleckchem and added to cells 4 hpi.

### Infection.

Cells were plated in 6-well plates at a density of 5 × 10^5^ cells per well and incubated at 37°C for 24 h. Cells were not infected (mock) or infected with *Δ*E3, *Δ*E1B, *Δ*E4orf3, *Δ*E4orf1-2, *Δ*E4orf1-3, or the *Δ*E4orf1 Ad at a Multiplicity of Infection (MOI) of 50 and incubated 1 h at 37°C with rocking every 10–15 min. Media were replaced with fresh media and incubation continued for 24, 48, or 72 hpi at 37°C.

### Flow cytometry.

DNA content was determined by flow cytometry. Cells were plated in 6-well plates and infected. Infected cells were collected at 24, 48, or 72 hpi in FACS tubes. Cells were washed with PBS and fixed with 70% ethanol overnight at −20°C. The fixed cells were then washed twice with PBS. After centrifuging, the pellet was stained with 500 μl of FxCycle^TM^ PI/RNase staining solution added to each flow cytometry sample. The samples were incubated for 15–30 min at room temperature, protected from light. The DNA content of each cell was measured with a BD FACSVerse and analyzed with FlowJo.

### Immunofluorescence.

Cells were seeded on coverslips in 6-well plates at a density 5 × 10^5^ cells per well and incubated at 37°C for 24 h. Cells were infected and fixed 24, 48, or 72 hpi with 4% paraformaldehyde fixing solution (PFA) at room temperature for 15 min. The cells were then rinsed three times with PBS and permeabilized with PBS with 0.5% Triton X-100 (PBST) for 5 min at room temperature. Cells were washed three times with PBS and blocked with 5% normal goat serum and 1% BSA for 1 h at room temperature. The cells were then exposed to primary antibodies diluted in PBST. The cells were rinsed three times in PBS and exposed to Alexa Flour 488 nm goat anti-mouse or Alexa Flour 649 goat anti-rabbit fluorochrome-conjugated secondary antibodies (Life Technologies, Carlsbad, CA USA). The samples were washed three times with PBS and mounted with DAPI Fluoromount. Fixed samples were captured by using Nikon Ti-E-PFS inverted microscopy equipped with a 100 × 1.4 NA Plan Apo Lamda objective and outfitted with a Yokogawa CSU-X1 spinning disk unit and Andor iXon 897 EMCCD camera. The excitations were 405 nm, 488 nm, and 647 nm with an exposure time of 400 ms. Confocal images were acquired using NIS-elements. The levels of nuclear antigens were quantified using ImageJ. This was accomplished by selecting the ROI. DAPI in the 405 nm channel was used to select the nucleus from the rest of the cell. The ROI was applied to the other channels (green 488 nm or magenta 647 nm channels). Mean fluorescence intensity of five different fields were measured and the standard error of mean (SEM) plotted and compared. Three biological repeats were performed.

### Western blot.

Cells were infected. After 24, 48, or 72 h, cells were lysed in 1 × SDS sample buffer and 3% BME. Equal amounts of lysate were loaded into wells of 4–20% Tris-Glycine gels. Following electrophoresis, proteins were transferred from the gel to a nitrocellulose membrane. The membrane was blocked in PBS with 0.02% Tween 20 and 20% milk for 1 h at room temperature. After blocking, the membrane was incubated in appropriate dilutions of primary antibody in PBS with 0.02% Tween 20 and 10% milk for 2 h at room temperature. After washing three times in PBS with 0.02% Tween 20 and 5% milk for 5 min each time, the membrane was incubated with appropriate dilutions of secondary antibody in PBS with 0.02% Tween 20 and 10% milk for 1 h at room temperature. The membrane was washed three times, 5 min each. The membrane was exposed to a 1:1 solution of substrate luminal/enhancer solution and image captured using a LI-COR Odyssey Fc Imaging System (Lincoln, Nebraska USA).

### Luciferase assays.

HCT116/NF-κB reporter cells were seeded and infected in 24-well plates. Cells were washed with PBS and 1X lysis buffer was added to cells. The cells were transferred to 96-well plates and 100 μl of luciferase assay reagent were added to the cells. Plates were read immediately after addition of substrate solution by a luminometer. Luciferase expression was quantified as relative light units (RLU), normalized to readings of control wells, and expressed as relative NF-κB reporter activity.

### Statistical analysis.

The mean values are reported with the SEM. The two-tailed *t* test was used to determine the difference between the mean for groups of two and analysis of variance (ANOVA) for groups of more than two. *P values* ≤ 0.05 are considered significant.
