# Supplementary material for: Disruption of NBS1/MRN Complex Formation by E4orf3 Supports NF-κB That Licenses E1B55K-Deleted Adenovirus-Infected Cells to Accumulate DNA>4n
Source: Microbiol Spectr. 2022 Jan 12;10(1):e01881-21. doi: 10.1128/spectrum.01881-21 (PMC8754114; doi:10.1128/spectrum.01881-21)
Supplement: SUPPLEMENTAL FILE 1 — Supplemental material. Download SPECTRUM01881-21_Supp_1_seq11.pdf, PDF file, 2.5 MB [file spectrum01881-21_supp_1_seq11.pdf]

Figure S1

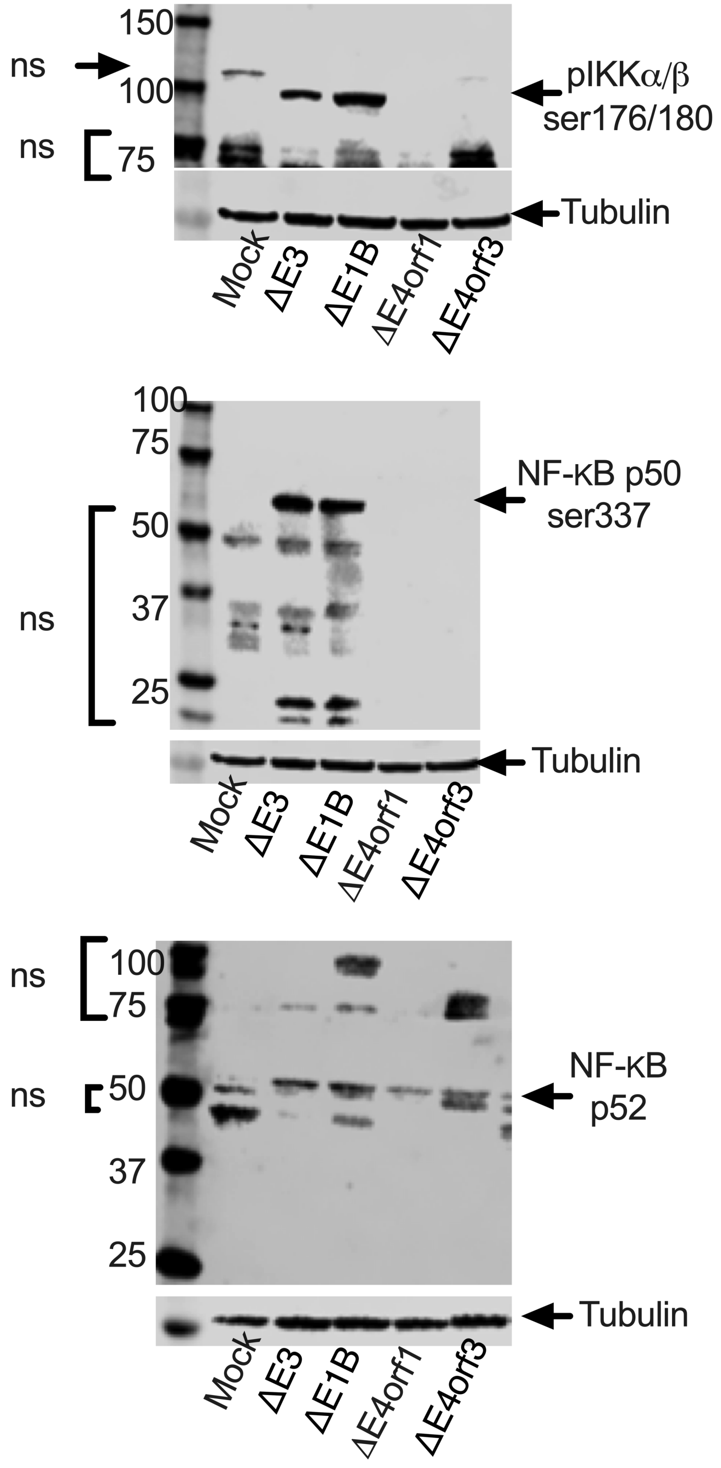

Figure S1 (related to Figure 3): Ad infections lead to canonical and noncanonical activation of NF-κB. Western blot images of phospho-IKKα/β (Ser176/180), phospho-p50 (Ser337) and p52 are shown. The extra bands are believed to be nonspecific and labeled as "ns" because they are either also in the mock or were not seen in every instance nor in other cell lines.

Figure S2

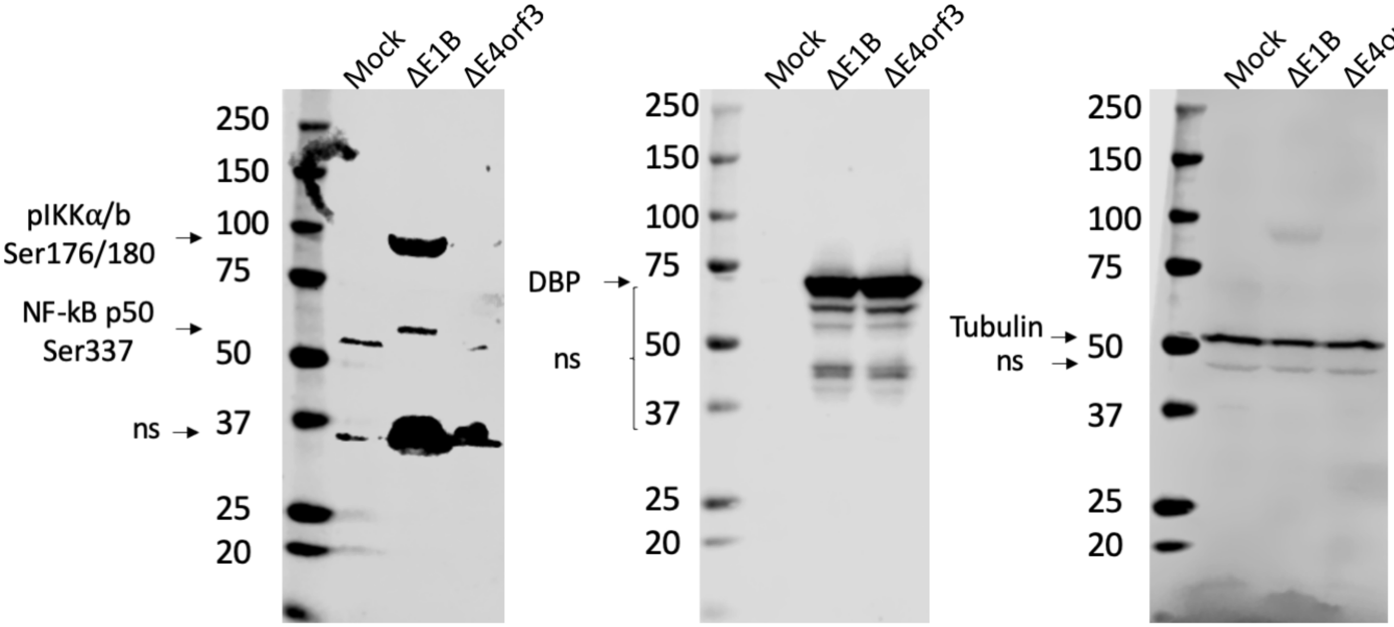

Figure S2 (related to Figure 4B): Ad-infections promote NF-κB transcriptional activity. HCT116-luc cells were not infected (mock) or infected with the indicated virus at a MOI of 50 for 48 hours. Western blot images of the static levels of phospho-IKKα/β (Ser176/180), phospho-p50 (Ser337), the infection control Ad DNA binding protein (DBP), and the loading control tubulin are shown. The extra bands are believed to be nonspecific and labeled as "ns" because they are either also in the mock or were not seen in every instance nor in other cell lines.

Figure S3

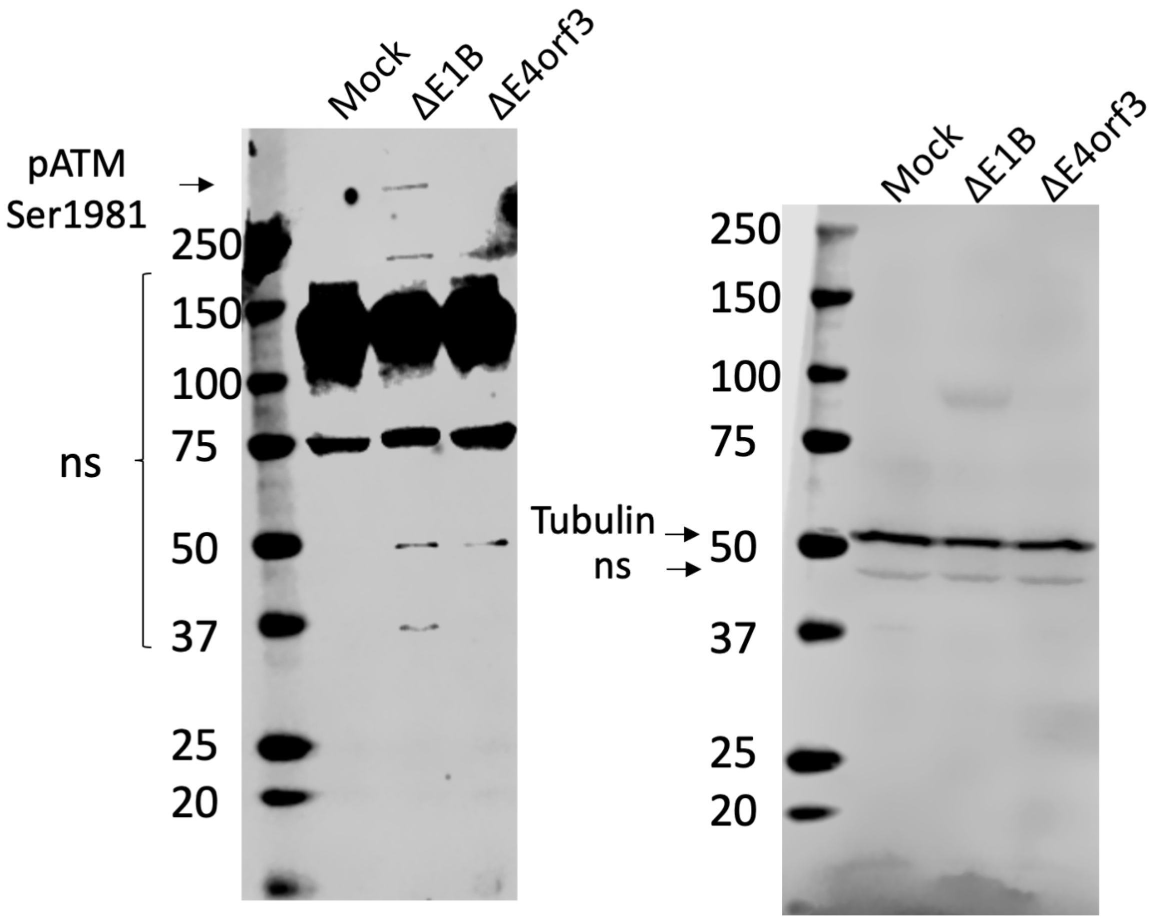

Figure S3 (related to Figure 6D): Inhibition of ATM kinase activity reduces DNA>4n and NF-κB activation in Ad-infected cells. Western blot image of the static levels of phospho-ATM ser1981 are shown in HTC116-luc cells. The extra bands are believed to be nonspecific and labeled as "ns" because they are either also in the mock, were not at the kda known for ATM, were not seen in every instance nor in other cell lines.

Figure S4

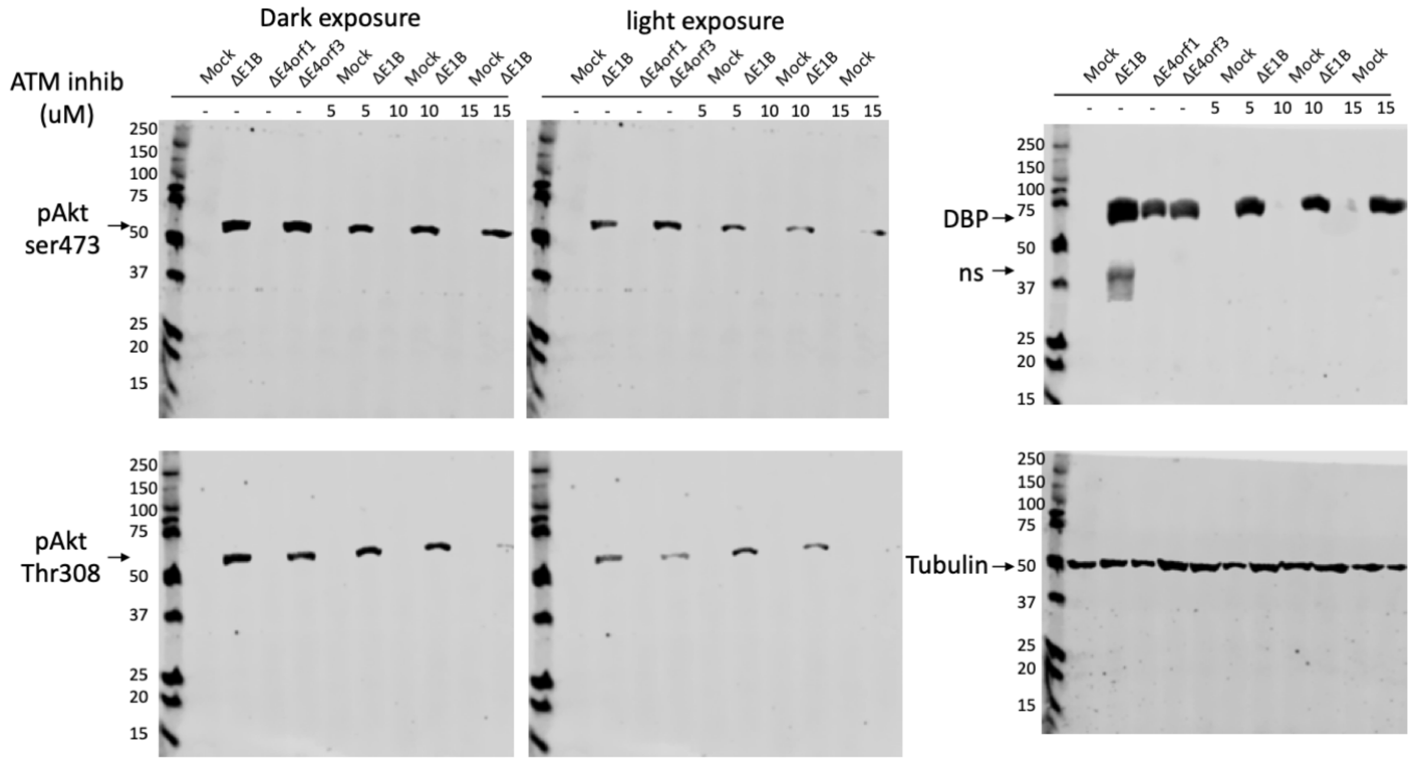

Figure S4 (related to Figure 6I): Inhibition of ATM kinase activity reduces DNA>4n and NF-κB activation in Ad-infected cells. HeLa cells were infected with the indicated Ad at a MOI of 50 and 4hpi treated with or without ATM inhibitor, KU60019 at increasing concentrations. Western blot image of the static levels of phospho-Akt (Ser473) and Thr308 are shown 48hpi. D = long exposure; L = short exposure. The static levels of the infection control Ad DNA binding protein (DBP), and the loading control tubulin, are shown. The extra band is believed to be nonspecific and labeled as “ns” because it was not seen in every instance nor in other cell lines.
